# An Isopotential Electron Titration Device Enables Real-Time Measurement of Transient Charge Transfer on Surfaces

**DOI:** 10.1021/acscentsci.5c01984

**Published:** 2025-10-24

**Authors:** Fang Xu

**Affiliations:** Department of Chemistry, 12346 The University of Texas at San Antonio, San Antonio, Texas 78249, United States

## Abstract

Abdelrahman and co-workers developed a device to measure transient charge transfer during hydrogen dissociation on a Pt surface.

## Graphical abstract

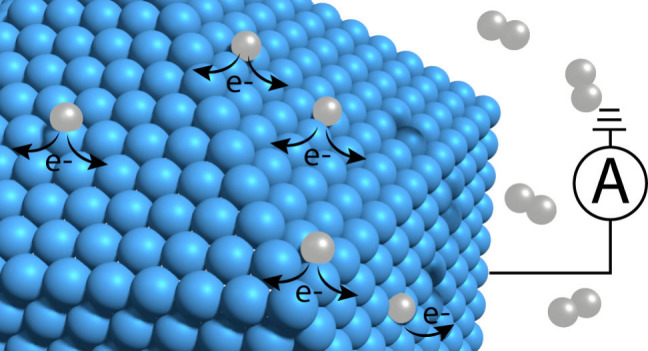

The whole universe is made up of atoms and molecules. At the nanoscale, all types of interactions involve electrons at the periphery of atoms. This is also true in heterogeneous catalysis. For a catalyst to function, molecules must adsorb onto the surface, react with each other to form products, and then desorb from the catalyst surface. Various physical and chemical processes, or elementary steps, in this sequence involve charge transfer between the two matters. In many cases, partial charge transfer or charge separation occurs, resulting in noninteger oxidation states. For example, single gold atoms undergo only partial oxidation when adsorbed on an Fe_3_O_4_(001) surface, as evidenced by a shift in the Au 4f X-ray photoelectron spectroscopy (XPS) peak.^1^

In catalytic reactions, both thermodynamics and kinetics play crucial roles in determining the overall catalytic performance. Take the hydrogenation reaction as an example: under typical pressure and temperature conditions, millions of hydrogen molecules in the gas phase collide with the catalyst surface. Some of these molecules simply bounce back without reacting, while others dissociate into bonded hydrogen atoms—an important intermediate that drives the reaction forward. At equilibrium, the net effect of hydrogen interacting with the catalyst surface is a constant concentration of adsorbed hydrogen atoms. Thermodynamically, the hydrogen molecules can indeed be “activated”. Kinetically, however, any change in reaction conditions, such as hydrogen partial pressure or temperature, will alter the concentration of active hydrogen atoms on the surface. Measuring the thermodynamically stable surface, particularly under vacuum conditions, is relatively straightforward using various X-ray spectroscopies^2,3,5^ and electron energy-loss spectroscopy.^4^

However, measuring dynamic charge transfer on a solid surface in real time using the mentioned common techniques remains highly challenging due to beam damage inherent in X-ray techniques.

Besides theoretical calculations, time-resolved charge transfer in solid materials has been experimentally measured using techniques such as transient absorption–reflection spectroscopy, time-resolved photoluminescence spectroscopy,^6^ and two-photon photoemission spectroscopy.^7^

In this issue of *ACS Central Science*, Abdelrahman and co-workers report the invention of a sandwich-shaped device composed of platinum/carbon on top of HfO_2_ and bulk p^++^-Si. This device successfully measures the charge transferred from adsorbed hydrogen to platinum as 0.19 ± 0.01% |e|/H.

The device responds rapidly to both the direction and nanoampere-level currents occurring between the Pt and p^++^-Si layers (Figure 1A).^8^ Based on a set of equations, the authors plot the dynamic net charge transfer resulting from hydrogen adsorption on the Pt surface (Figure 1B).

**Figure 1:**
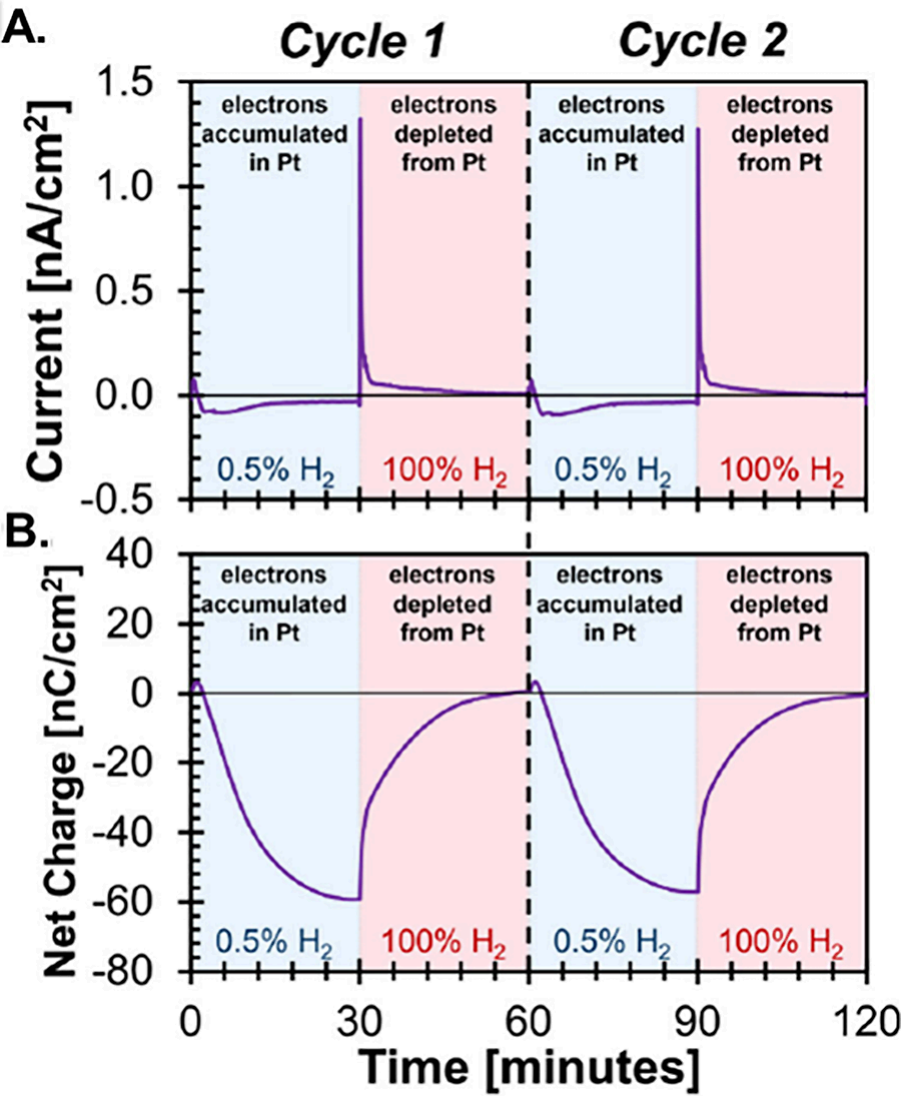
Current and net charge measured by the isopotential electron titration device during hydrogen adsorption on Pt. Reproduced with permission from ref 8. Available under a CC-BY 4.0 license. Copyright 2025 Justin A. Hopkins, Benjamin J. Page, Shengguang Wang, Jesse R. Canavan, Jason A. Chalmers, Susannah L. Scott, Lars C. Grabow, James R. McKone, Paul J. Dauenhauer, and Omar A. Abdelrahman.

The device was carefully fabricated and characterized, including noise-level determination, failure analysis, theoretical comparisons, and verification by two independent research teams to ensure the reliability of the transient measurements. The direction and shape of the observed peaks provide valuable information, such as the charge-transfer direction, rate, and equilibrium adsorption coefficient. Furthermore, temperature and pressure effects were examined by heating up to 400 °C and varying the partial pressure of hydrogen.

The isopotential electron titration device provides a reliable method for studying the dynamic net charge transfer between adsorbates and a catalyst surface, a process that is critical for understanding fundamental catalytic reaction mechanisms.

Unlike traditional techniques that require an ultrahigh-vacuum setup to obtain electron-related measurements, such as work function and oxidation states of solid materials, the isopotential electron titration device operates under more accessible conditions, thereby lowering both the knowledge and cost barriers to its use and maintenance. When combined with theoretical calculations and spectroscopic analyses, this invention offers a powerful alternative approach for identifying field-responsive catalysis on solid surfaces.
